# Epidemical and Local Influences on Disease

**Published:** 1856-04

**Authors:** 


### EDITORIAL AND MISCELLANEOUS.

### EPIDEMICAL AND LOCAL INFLUENCES UPON DISEASE.

In looking over the medical periodicals of the present day, it is not unusual to find the most conflicting views in regard to the treatment of our most common diseases—the remedial agents used, and apparently with the same success, being directly opposite in their character and action. To illustrate: we may see a communication from an eminent and reliable physician, of one section of our State, who treats pneumonia, for example, with the lancet and tartar emetic, with the most satisfactory results, while, perhaps, in the same journal, we have a communication upon the treatment of the same disease from another location, and from a physician equally eminent and reliable, who cures the disease with opium, quinine, &c., protesting loudly against the use of the lancet and tartar emetic, considering them unreliable and unsafe remedies.

Nor does this difference of opinion alone exist in regard to the above mentioned disease, but extends, perhaps, even to a greater extent, in some others. How shall we account for this discrepancy of opinion? Shall we distrust the reporters, or, for a moment, doubt the correctness of the great principles of our science? Should we not, rather, attempt to account for those apparently contradictory results by referring them to influences, the existence and importance of which have been long since satisfactorily demonstrated? I allude to epidemical and local influences.

It was, I believe, the illustrious Sydenham who first attempted to impress the profession with the great importance of considering the influence of epidemics in the treatment of disease, or in other words, the connection which exists between the "distemperature of the air," and the type or tendencies of the prevailing disease.

There is no truth in medicine, in our opinion, a proper appreciation of which is of more importance to the practitioner of medicine than that of epidemical influences. Every physician who has given this subject a moment's reflection, must admit that the character, force, &c., of our most common diseases are continually undergoing a change, and that the treatment, if at all successful, must be regulated by the influences which are brought to bear upon them.

To hear, as I have often done, and from the most intelligent physicians of this section of our State, a detail of the peculiar character of autumnal fever as it existed twenty-five to thirty years ago, and the heroic treatment which was then found successful, we are fully impressed with the great change which has taken place in the character, force and tendencies of this disease since that date. Venesection and drastic cathartics, and the latter continued for several successive days, constituted an important item in the treatment of autumnal fevers at that time. That such a course of treatment would, at present, be decidedly injurious, if not fatal in the majority of cases, no one will deny, but that this is an evidence that it accomplished no good then, but was generally attended with injurious consequences, I am not prepared to agree. And in this connection I would remark, that the heroic treatment of the immortal Rush was, perhaps, in a number of diseases, less censurable than we of the present would imagine.

But to more forcibly illustrate the object of this article, I would call attention to the epidemic of typhoid fever which prevailed to an alarming extent in middle and western Georgia, in 1850-51 and '52. During the prevalence of this epidemic, and, to some extent, since it has subsided, all acute diseases appear to have been more or less impressed with its peculiar type. All who were engaged in the practice where this epidemic prevailed, will agree that, in the treatment of acute inflammatory affections, unconnected except by epidemical influences with the prevailing fever, great caution was necessary in the use of active remedies—remedies that were usually relied upon before the appearance of the epidemic; that, if such remedies were used and persisted in, which was, unfortunately, often the case, the accompanying fever would assume a low typhoid form, from which the patient would be weeks, and sometimes even months, recovering. Nor was it always necessary to deplete the patient to bring on this state, as in quite a number of cases the symptomatic fever, from the first, assumed the asthenic type—demanding, for treatment, stimulants rather than the lancet. This tendency also extended to the autumnal fevers, marking them to such an extent, that it was often extremely difficult to determine their precise character.

That venesection and tartar emetic are less used now in acute inflammatory affections, and justly so, than they were previous to the epidemic, is a fact beyond dispute, but not an evidence that they were at that time improperly employed, or that they have at present fallen into disuse from that caprice which regulates our social relations.

We have thus alluded to autumnal fevers as they existed many years ago, and briefly to the more important features of the recent epidemic, as it goes far to demonstrate, as well as confirm, the position with which we set out: that diseases, even in the same locality, and presenting the same pathological changes, are under different epidemical influences, so changed in type, tendencies, &c., that remedial agents which will at one time cut short the disease, will, under other circumstances or influences only tend to increase the existing difficulty.

Again, local influences, of which we have as yet said nothing, should not by any means, be passed by as unimportant in connection with the treatment of disease. We feel assured that a want of proper appreciation of this feature of our subject, has often resulted in the most serious errors.

In acute inflammatory affections, we would not think of treating with the same activity, a subject who had for years lived in a miasmatic district, and been subject to all its contaminating influences, as we would the hale mountaineer. In miasmatic districts, then, where the system is to a certain extent impressed with its peculiar influence, we should have great care in the use of active remedies, even in acute affections. Many physicians who have, for years, practiced their profession in mountainous districts, where every face indicated health and vigor, have been led astray by their success for a series of years, in treating acute inflammations with the most heroic remedies—imagining that all acute inflammations, under whatever circumstances or influences presented, must be combatted by the lancet and like antiphlogistics. A similar error might result from a successful experience in low malarial districts.

Much more might be added in illustration of this important subject, but as this article has already been extended to a much greater length than was at first intended, we will defer any further remarks at present, and in conclusion, would urge upon the profession a proper consideration of this subject in their reports. In communications upon the treatment of disease, let us have with it the peculiarity of location, the charactic of the last epidemic, and the general type, tendencies, &c., of disease. When this is done, and not until then, may we expect a proper appreciation of the importance of epidemical and local influences.

